# Care Coordination Experiences of Whole Families Caring for Children With Medical Complexity in Rural Areas

**DOI:** 10.1111/hex.70793

**Published:** 2026-08-03

**Authors:** Anneliese de Groot, Karen Hutchinson, Amy Hickman, Raghu Lingam, Jeffery Fletcher, Hayley Smithers‐Sheedy, Yvonne Zurynski

**Affiliations:** ^1^ Australian Institute of Health Innovation Macquarie University Sydney Australia; ^2^ School of Population Health University of NSW Sydney Australia; ^3^ Central Coast Local Health District, NSW Health Gosford Australia; ^4^ Flinders University Adelaide Australia; ^5^ School of Clinical Medicine University of NSW Sydney Australia; ^6^ Bond University Gold Coast Australia; ^7^ Faculty of Medicine and Health, Cerebral Palsy Alliance Research Institute The University of Sydney Sydney Australia

**Keywords:** care coordination, children with medical complexity, family, family‐centred care, integrated care, paediatric, rural healthcare

## Abstract

**Background:**

Family caring roles create significant impacts on individual family members' physical, mental and social wellbeing, and can disrupt family financial stability, relationships and functioning. The perspectives of family members other than mothers are often omitted from research, reducing understanding of the broader impacts of supportive interventions such as care coordination.

**Objective:**

This study aimed to understand how multiple family members experience the impacts of caring for children with medical complexity (CMC) in a rural setting in Australia, and how paediatric care coordination influences their experiences.

**Design:**

This qualitative study involved thematic narrative analysis and multimember comparison to identify themes within and across families, which were then mapped against the socioecological model.

**Setting and Participants:**

Members of 13 families of CMC receiving a nurse‐delivered care coordination intervention, the Rural Kids Guided Personalised Service, participated in semi‐structured interviews, including mothers (*n* = 12), fathers (*n* = 7), siblings (*n* = 3), CMC (*n* = 2) and an aunt (*n* = 1).

**Results:**

Five themes were generated, including: Caring for CMC in varied family contexts, Coordinating care to support family strengths, Accessing appropriate care when living in a rural area, Impact on the whole family (including two subthemes: Family relationships and Enabling support within and for families) and Quality care closer to home.

**Conclusions:**

These findings are relevant for future implementation of integrated care models, and can inform the development of expanded models of care that address broader family health and social needs. Future service evaluations would benefit from inclusion of multiple family members, rather than only mothers, to allow a more comprehensive understanding of how best to support whole families.

**Patient or Public Contribution:**

Parents with lived experience of caring for CMC in rural areas sat on the steering committee guiding the implementation and evaluation of RuralKidsGPS, alongside clinicians, Aboriginal health workers and advisors, health managers, researchers and policy officers.

## 1 Introduction

Family carers form the backbone of health and social care systems around the world, supporting and nurturing children, elders and family members with additional medical and social needs [1]. Children with Medical Complexity (CMC) have one or more chronic, complex diagnoses, such as cerebral palsy or rare diseases, requiring a high level of specialized medical care, which may include as many as 15 specialists [2, 3]. Family members are usually CMCs' core care providers [4], and they address CMCs' daily care needs while also coordinating complex healthcare across multiple health sectors [3]. As a result, family caring roles create significant impacts on individual family members' physical, mental and social wellbeing, and can disrupt the whole family's financial stability, relationships and family functioning [5, 6, 7].

The whole family is a collective of multiple family members caring for CMC. Family is defined as individuals bound by significant relationships involving reciprocal care and trust [8], which can include a diverse range of family configurations including same‐sex and single parent families, and those in which extended family members fulfil parenting roles [9]. In research exploring the impact of caring on families, family can refer to the whole family, collectively, or only one family member, commonly the mother [10]. While some studies have explored caring experiences amongst fathers [11], siblings [12] or extended family members [13], the perspectives of family members other than mothers are often omitted from research.

Including whole families in service evaluations is necessary to understand the broader impacts of supportive interventions. Much of the work of caring for CMC is comprised of healthcare navigation and coordination tasks [14, 15]. Family carers must learn how the healthcare system functions to ensure they access the care required for their child [16, 17]. Because the healthcare system is designed around healthcare provider and administrator needs rather than complex child or family needs, navigation can be time‐consuming and stressful [18, 19], impacting the whole family [20]. Care coordination services are often implemented to support family carers to manage CMC healthcare, and are reported as highly beneficial for the quality of care received and families' experiences of care [21]. However, care coordination evaluations are typically conducted with only one family carer [22, 23], rarely including the perspectives of multiple family members [24]. This leaves a gap in our understanding of how care coordination, or other supportive interventions, impact the family as a whole both in urban and rural settings.

Families of CMC regularly travel to access healthcare from tertiary hospitals, medical specialists and allied health services, incurring high financial and productivity costs and disrupting schooling, employment and family routines [25, 26]. Travel to healthcare services is greatly increased when living in rural or regional areas [27], and can negatively impact family carers' quality of life [23, 28]. Whether supportive interventions can reduce the impact on families of CMC living distant to services is not currently well understood. Therefore, this study aimed to understand how multiple family members experience caring for CMC in a rural setting, and how paediatric care coordination influences their experiences.

## 2 Methods

This narrative thematic inquiry was informed by a pragmatic and strengths‐based approach, identifying families' and healthcare providers' strengths rather than positioning families caring for CMC as problematic or vulnerable [29]. Each family shared rich stories to organize and make sense of their experiences [30]. Interrogating these narratives generated knowledge about how services can address the issues faced by families, to inform future interventions [31].

### 2.1 Context

This study was conducted as part of the implementation evaluation of a rural paediatric care coordination service [32]. Based on a successfully trialled metropolitan model of care [25], the Rural Kids Guided Personalised Service (RuralKidsGPS) was implemented and evaluated in four local health districts (LHDs) in the state of New South Wales (NSW) between 2021 and 2024 [33]. A steering committee guided the implementation and evaluation of RuralKidsGPS, including parents with lived experience of caring for CMC in rural areas, clinicians, Aboriginal health workers and advisors, health managers, researchers and policy officers. The LHDs comprised rural and regional areas, with long travel distances to access tertiary children's hospitals. Sometimes the nearest tertiary children's hospital was located outside of NSW in another state jurisdiction, limiting capacity for health record sharing because of different electronic platforms and different privacy laws across state borders.

The care coordinators delivering RuralKidsGPS were experienced specialist paediatric nurses based in the rural LHDs. The personalized child and family‐centred care coordination spanned multiple care teams, health facilities and linkage with the social care and disability sectors, in addition to streamlining appointments and providing a central point of contact for families with CMC [32]. The first author (AdG) was a locally embedded researcher within one of the LHDs, working alongside the care coordinator. In this role, AdG recruited family carers and administered web‐based and phone surveys at multiple timepoints for the impact evaluation, before commencing a Doctor of Philosophy degree supervised by the implementation evaluation team. Thereby, AdG had started gaining insight into families' stories through the implementation evaluation before commencing this standalone study [34]. Ethical approval for this study was obtained from the Sydney Children's Hospital Network Human Research Ethics Committee (2021/ETH01055).

### 2.2 Recruitment

RuralKidsGPS was available for CMC aged 0–18 years with a medical diagnosis expected to last 12 months or more, and high service use [32]. Eligibility was based on CMC's complexity and need rather than specific diagnoses. Between 6 and 12 months after CMC's RuralKidsGPS enrolment, parents were invited to an interview and chose whether to also invite additional members of their family. All family members provided voluntary informed consent, electronically using Adobe [35]. An age‐appropriate Participant Information and Consent Form was provided for child participants aged 7–18 years. All participants had the opportunity to ask questions of the locally embedded researcher in each LHD to ensure they understood the study. Families were not invited if flagged by the care coordinator as being in a state of crisis or overwhelmed by their caring circumstances.

### 2.3 Data Collection

The semi‐structured interview guide was developed to understand broad experiences with the care coordination service, including open‐ended questions about caring for CMC, interacting with health and support services across multiple sectors, impact on individual family carers and the whole family, and family wellbeing. Interviews were conducted in‐person at hospitals or community health centres, or over Zoom [36], by either one or two interviewers (KH, AdG). The second author (KH) had extensive prior experience interviewing family carers and people with disabilities, including children and young people, and provided training for the first author (AdG), who had established rapport with many of the participants through prior contact. Debriefing after interviews involved both interviewers with additional members of the research team [37]. Reflexive journaling was undertaken throughout the research process to acknowledge researcher biases that might influence data collection or interpretation [38].

Interviews were recorded through Zoom [36] or using a digital audio recorder, and transcribed verbatim by either a professional transcription service, DAATS [39], or using the in‐built transcription function in Microsoft Word [40]. Transcripts were checked by the first author (AdG) to ensure accuracy, and were de‐identified prior to analysis.

Supporting data were included from a free‐text item in an adapted survey measuring non‐medical out‐of‐pocket costs incurred by families travelling to access healthcare [41]. This survey was administered at up to three timepoints, over the phone, for the impact and economic evaluations of RuralKidsGPS. This text was entered as accurately as possible to reflect each respondents' verbal response to the prompt ‘are there any other details relevant to the logistical, administrative or financial impact of the care for your child when admitted to hospital or attending a healthcare appointment, to you and/or your family network, that we have not captured, and that you wish us to know?’ This data added details to some families' narratives and confirmed the significance of stories arising repeatedly across time points.

### 2.4 Analysis

A hybridized approach incorporated thematic narrative analysis and multimember comparison to interpret families' stories [30, 42]. After initial familiarization with each transcript, the stories were identified according to their perceived importance to the speaker [30]. This was recognized in events that required explanation, referred to as trouble, and was identified by significant departures from the interview questions, and stylistic, grammatical, paralinguistic and lexical features [43].

**Figure 1:**
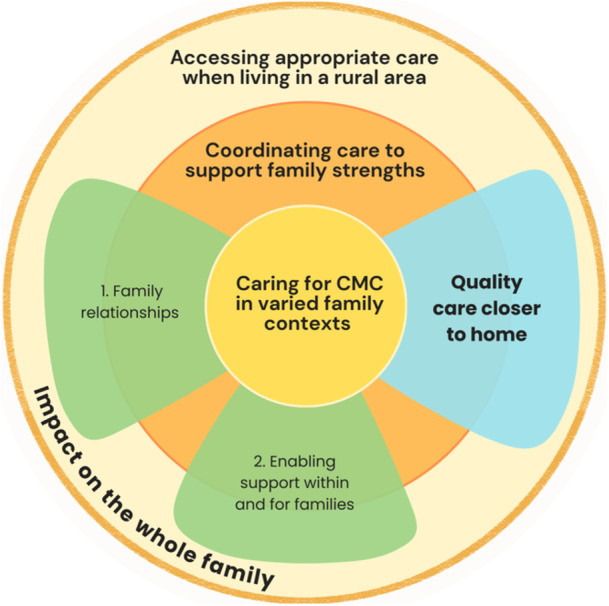
Themes and sub‐themes: Experiences of whole families caring for children with medical complexity (CMC) in rural areas.

The first author identified themes within stories, which were iterated and refined through multiple discussions with the broader research team and with researchers and care coordinators from the larger RuralKidsGPS team. Stories and themes were compared between multiple family members within each family unit to understand similar and varied perspectives and experiences [42]. This comparison generated increased depth of insight supporting the interpretation available to the authors [42]. Themes were organized using NVIVO 20 [44]. The relationships between themes (Figure 1) were identified and mapped using the socioecological framework to explore the complexities of families' ecology and their connections with the health system [19, 45]. Using this framework highlighted the interactions between family members (microsystem), between families and healthcare providers (mesosystem) and the broader contextual influences (macrosystem) [45].

## 3 Findings

Sixteen interviews were conducted with members of 13 families, including 12 mothers, seven fathers, three siblings, two CMC, and one aunt. Six interviews were conducted jointly with two or three family members, and 10 were conducted with individuals. Mothers led the conversation in group interviews, reflecting a high level of care experience within each family. All families spoke English at home and lived in areas of moderate to high disadvantage according to the Index of Relative Socio‐economic Advantage and Disadvantage [46], where one is most disadvantaged, and five is most advantaged (mean = 1.9, range 1–3). CMC in the 13 families had a median age of 3 years old (range: 1–16), were predominantly male (*n* = 8, female *n* = 5), and had diagnoses in a median of two different diagnostic categories (range: 1–6), most commonly neurological (*n* = 7), cardiovascular (*n* = 6) and digestive (*n* = 5) [47]. Families lived distances of 100–499 kilometres away from their tertiary hospital (modal class: 100–199). In five families, fathers, siblings and extended family members were willing to be interviewed but were unavailable during the study period. In three families, mothers declined to invite additional family members, including fathers, male siblings and extended family members, citing that they were not involved in care or too busy with employment or sports. The family systems of each participating family can be seen in Table 1.

**Table 1:** Members of each family unit and codes for participating family members.

ID	Members of family unit	Participant codes^a^
1	Mother, father, CMC, sibling	(M1, F1, S1)^b^
2	Mother, father, CMC, sibling	(M2, F2)
3	Mother, father, CMC, 2 siblings	(M3, S3, C3)
4	Mother, father, CMC, 2 siblings	(M4, S4)
5	Mother, father, CMC	(M5, F5)
6	Mother, father, CMC, sibling	(F6, C6), M6
7	Mother, CMC, 2× siblings	M7
8	Mother, father, CMC	M8
9	Mother, father, CMC, 1× siblings	M9, F9
10	Mother, father, sibling, CMC	M10
11	Father, CMC	F11
12	Mother, father, CMC, 2× sibling	M12, A12
13	Mother, father, CMC, 2× siblings	M13

a M = mother, F = father, S = sibling, C = child with medical complexity, A = aunt.

b Brackets indicate joint interviews with multiple family members.

### 3.1 Summary of Findings

The findings from families' stories formed five core themes and two subthemes (Figure 1). The first theme (*Caring for CMC in varied family contexts*) centred around the experience of each family meeting CMC needs according to their own and their child's particular circumstances. Theme two (*Coordinating care to support family strengths*) examined how care coordination connected families to rural health services and provided individualized support. Theme three (*Accessing appropriate care when living in a rural area*) explored challenges of travelling to access care. Theme four (*Impact on the whole family*) explored the impact of caring on various family members in their caring roles. Theme four included two subthemes, *Family relationships* and *Enabling support within and for families*. The fifth theme (*Quality care closer to home*) explored families' stories of healthcare experiences before and after receiving care coordination, highlighting ways in which the RuralKidsGPS service reduced family stress by advocating for CMC when accessing local emergency healthcare.

Figure 1 illustrates relationships between the themes from families' stories. Families caring for CMC inhabit the central circle as the microsystem (Theme one). The care coordinators, as the mesosystem, encircled families and formed the intersection between families and their numerous interactions with other branches of the health system (Theme two). The outer ring represents families' rural context as the macrosystem that influenced families' experiences, for example, inequity in accessing required health services from rural areas (Theme three). Themes four and five spanned the micro, meso and macrosystems.

### 3.2 Caring for CMC in Varied Family Contexts

Experiences of caring for CMC varied according to each family's circumstances. The nature and severity of CMC diagnoses influenced families' day‐to‐day experiences, such as the ability ‘to go out as a family’ (M13). Families also received different levels of support and coordination, largely based on CMCs' diagnoses and available services for that diagnostic group, prior to receiving RuralKidsGPS. Families of children with more common diagnoses, or a prognostic pathway such as palliative care, had greater support, including some integration of their local and tertiary healthcare, which improved their experiences with care closer to home, compared with families of children with undiagnosed or rare conditions. Families' experiences also varied according to the recency of CMCs' diagnoses, as they gained experience with caring for CMC over time (see Table 2).

**Table 2:** Narratives demonstrating support and coordination pathways and caring experiences.

Narrative	Quotes
Some connection between tertiary and local services for child with common diagnosis.	[Tertiary hospital] was very good at trying to patch us in to local people. Like if something happened locally, we knew who could help on the ground here … and so they tried to make sure that [Nearby Town] hospital knew [Child's] file. (M6)
Newborn with complex rare condition had poorly coordinated discharge from the neonatal intensive care unit in a metropolitan hospital, home to a rural area, without appropriate support.	[Nurse] just came out with a piece of paper and said ‘[Child] has all these changes to his brain you need to go and get a really good pediatrician’. Didn't give me a referral. ‘You need to go and see a neurosurgeon. We don't have them at hospital here you'll have to find one yourself’. So I was then in the position where I've then walked out of the hospital‐‐ in the car park, Googling, going ‘What am I supposed to do now? (M4)
The family was in the early stages of crisis, adjusting to their caring roles while managing multiple demands. They faced challenges balancing responsibilities and coping with emotional strain, including conflict, overwhelm, vulnerability and uncertainty about the future.	My husband … [said] ‘I'm not coping with the extra load because you're unwell and how much I have to carry [Child] and do this and do that and blah, blah, blah’. And that I mustn't understand how full his days are. And I blew up because I'm like ‘Are you f***ing serious? (laughs)… I do not include you in the five emails a day I get about things that I've gotta follow up for [Child], or the report I need to forward on, or the evidence the NDIS is requiring to justify some other thing, or the invoice I've gotta send to the plan manager’ … We are using way more support hours than we have at the moment, and we don't know what that looks like long‐term. We are so both incredibly aware how much time we need for ourselves and get none, and it is a cause of our mental health totally plummeting. But I don't know how to fix it. (M13)
The family had reached a level of familiarity with caring and was consciously normalizing their family experience.	[Child] goes to daycare twice a week which is good and you know she does Physie [exercise] with [Sibling] and she's pretty good at that (laughs) so yeah, just trying to‐ just normalize things as much as we can within‐ I guess within reason and that but definitely the fact that you can‐‐ have solid foods now and‐ moderately thick fluid [through feeding tube] definitely‐‐ definitely that was a bit of a life changer I think ‘cause yeah we were struggling before that. (F9)

Abbreviation: NDIS, Australian National Disability Insurance Scheme (NDIS, 2023).

Many stories illustrated families' willingness to work as a team, and families with established shared caring arrangements felt they had ‘things under control, fairly well’ (F1). However, even when families shared care, mothers still held the greatest responsibility for care, and attended the majority of healthcare appointments, as one father explained: ‘[Mum] handles a lot of this stuff and she's really good at being on top of it and she‐ I think she likes being … that person taking the lead with it’ (F9). Families with multiple family members sharing caring as a team were more effective in supporting each other, meeting their collective wellbeing needs and alleviating some of the stress and impacts of caring (Table 3).

**Table 3:** Sharing the experience of caring as a family team.

Narrative	Quotes
Both parents understand and participate in care for CMC collaboratively.	F5: [When there is an emergency] We take a big breath in, and then we just look into it, and just go for it, and just – yeah, whatever needs to be done, just … work it out. M5: Yeah, whenever something starts going wrong at home and we feel the need to call an ambulance, we've got our roles, and our responsibilities pretty much down pat.
Father is not actively involved in caring, with the mother receiving support primarily from extended family members.	In terms of involvement in [Child]'s life, myself and my mum do tenfold compared to what [Dad] does. (A12)
Mother acknowledges her role in not involving the father more in care for CMC and sharing her experiences with him	I feel a bit alone ‘cause it is just me … I [hold 100% of Child's care] and it's not my husband's doing. It's my doing. … [Dad] always knows what's going on. He's a really supportive person but I feel like it's on my shoulders and I'm‐ I am the organizer of our family and those things. (M10)
Parents are supported by extended family, and each other, to take time for themselves and avoid burnout	[Grandmother is] on top of it in regards to you know how to feed [Child] and timings and that and we always make sure we're giving her like any updates in the care plan. We [Mum and Dad] always encourage each other to find time to like do like our own exercise or something like that … just being aware of what each other's needs are. (F9)

Abbreviation: CMC, children with medical complexity.

Families who had greater employment flexibility were able to share caring tasks, particularly attending appointments. Many families described changing their employment circumstances to increase flexibility, either to enable fathers to attend appointments, for mothers' respite (see Table 4), or for both parents ‘to get to the most important of the appointments that we attend’ (M13). In several cases, fathers' responsibilities as farmers limited their ability to support mothers, as they needed to focus on maintaining their farm (Table 4).

**Table 4:** Flexible employment.

Narrative	Quotes
Father changed to a more family‐centred workplace to enable flexibility to attend appointments, for mother's respite	F2: I've just started a new job … and being able to take time off and organizing work around our stuff, so I think it is a lot better … M2: Yeah, whereas [Dad]'s old work, it was always a more‐‐ F2: It never really worked in our favour, it was more work than family unfortunately, but in a better place now. So take more time off. M2: So [Dad]'s been able to do things like … take [Child] to the speech and dietician.
Father's options to attend hospital along with mother and CMC were limited due to running a farm and therefore having no option to take leave.	[Dad] could only really leave the farm for a couple of days at a time … he does what he can, but I guess he's also, you know, there's things that have to keep happening on a farm. I guess unlike other sort of jobs where you can just drop everything and someone else will all carry on for you. (A12)

### 3.3 Coordinating Care to Support Family Strengths

Care coordination delivery varied according to each families' needs and strengths. Almost all families emphasized the importance of receiving care coordination and support early, suggesting for families ‘to be picked up as early as possible … because … you just don't really understand the full scope because you're not a doctor, you're just a parent’ (M4). Over time, parents develop a high level of health literacy and self‐advocacy skills, illustrated by one parent describing how ‘the nurse was just like, “Do you have a medical background?” And I was like, “No, I have a kid with [disease], can we just chuck the SATS [oxygen saturation] probe on this kid now?”’ (M2). Yet, even families with many years of experience navigating CMC healthcare and advocating for CMC's needs within the health system acknowledged the value of the additional support they received through RuralKidsGPS. Participants described how the care coordinators helped them overcome some of the barriers they encountered when interacting with the health system, such as ‘not really understanding where to go or who to talk to’ (F9), depending on their level of experience with caring.

### 3.4 Accessing Appropriate Care When Living in a Rural Area

**Figure 2:**
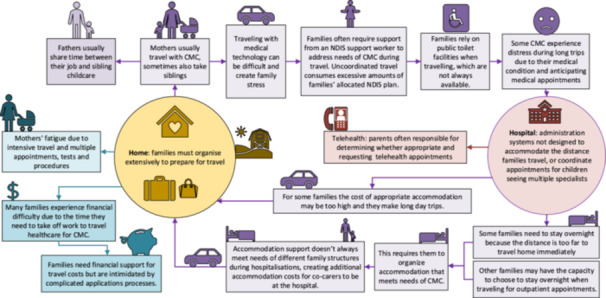
Accessing appropriate care when living in a rural area (supporting quotes in Supporting Information S1: Appendix A). CMC—children with medical complexity, NDIS—Australian National Disability Insurance Scheme (NDIS, 2023).

All families described stress and challenges related to reduced availability and quality of local services in their regional or rural area, explaining that their areas were ‘definitely lacking … with specialist services’ (F9). The specialist paediatric services required by CMC were often only available in tertiary hospitals in metropolitan areas, in most cases several 100 km away. All CMC required hospital admissions to tertiary hospitals in the city, for assessments and diagnosis, surgical procedures or health crises, which sometimes involved ‘[one‐]week to three‐week stays’ (M9). They also travelled to the city to access routine sub‐specialist paediatric outpatient appointments ‘sometimes once a month or once every two months’ (M9). Frequent long‐distance travel to access healthcare impacted whole families and created numerous challenges for families (Figure 2 and Supporting Information S1: Appendix A), who described it as a ‘pretty big thing [effort] every time we do it’ (M2), as captured in theme four.

Care coordination reduced the impact of distance by communicating with tertiary hospital administration to coordinate appointments to reduce unnecessary travel, particularly for frequent outpatient appointments: ‘[Care coordinator] would ring up and say “can we get all these appointments over … a two‐day period” and then we could just go down for one trip’ (M9). When interacting with the tertiary hospital administration staff who scheduled their outpatient appointments, rural families perceived ‘a presumption you're around the corner, you live local’ (F1); and wished ‘they [administrators] could be a little bit more lenient to those people that are traveling such vast distances’ (M4). Families appreciated the reduced financial impacts of travelling less and the RuralKidsGPS coordinators also facilitated families' applications for financial support, which often fell ‘off the “things to do” because it meant I had to chase paperwork and fill this thing out’ (M13).

### 3.5 Impact on the Whole Family

Caring for CMC impacted each individual family member differently, disrupting ‘the whole family as a unit’ (M4) with families saying, ‘we've been used to the disjointed family as far as everything we've gone through’ (F1). In addressing the needs of the whole family, parents typically adopted traditional gendered roles, usually the mother becoming the key carer while the father became the breadwinner. However, exceptions included single‐parent or co‐parenting families. Several families reported that both parents worked part‐time and shared care, or the mother worked full‐time rather than the father, who was the main carer.

Travel to access tertiary healthcare for CMC was commonly undertaken by mothers alone with CMC (Figure 2): ‘it was just me that could go … from when [Child] was two weeks old so that was two weeks post‐caesar [caesarean‐section] for me (laughs) which was‐‐ yeah tough ‘cause I was sleeping on a chair’ (M9). Many mothers experienced trauma, particularly during initial CMC hospitalizations. Some parents shared this experience, with fathers acknowledging mothers' trauma from ‘everything she [Mother] had to go through’ (F9) or experiencing trauma alongside mothers, when ‘our PTSD [post‐traumatic stress disorder] was very palpable’ (M8). In other cases, mothers felt that other family members didn't fully appreciate the intensity of their lived experiences, leading to feelings of isolation: ‘my husband … wasn't listening to me… he was basically saying, “oh, you're just doing it [parenting Child] wrong”’ (M12). Some mothers experienced mental breakdown and debilitating physical health issues due to the stress and physical strain of caring for CMC, which required hospitalization, including suspected seizure and back injury. COVID‐19 restrictions preventing multiple carers from attending hospital with CMC increased the intensity of mothers' experiences as it ‘fueled the “[Mum] is doing it on her own and us boys are chilling at home” … It just disconnected us even more’ (F1).

Fathers usually remained at home while mothers travelled to the hospital, maintaining employment as they felt responsible to ‘provide for the family’ (F9). Several fathers used the word ‘helpless’ (F1, F9) to describe their incapacity to support mothers during trips to tertiary hospitals. Fathers often shared childcare for siblings with extended family members while at home, or travelled between hospital and home to support mothers ‘when he could’ (M9). In some cases, fathers had to ‘go away and work so that we can afford to cover both wages’ (M4) when mothers couldn't return to work or so that mothers could reside closer to specialist healthcare services with CMC ‘until we felt it was safe’ (M10) to return home to a remote area. This created extended periods of separation between the father and the rest of the family, resulting in the father's heightened vigilance for children's safety when at home, with the mother noticing ‘he [Dad] seems to get up through the night and he checks on them [children] a lot’ (M4). One father assumed greater caring duties when the mother became overwhelmed, having ‘a hard time trying to wrap her head around everything (F11).

Siblings were frequently described as being ‘shipped off’ (M1) to stay with extended family for long periods during CMCs' health crisis periods. In many families, siblings also spent considerable time ‘traveling between home and [tertiary hospital]’ (M6) to visit CMC. However, siblings were also sometimes ‘visibly upset’ (M6) about CMCs' illness and could become anxious about ‘scary’ (S3) medical environments, resulting in long‐term physiological symptoms of stress, such as ‘withholding his bowel motions … he had a huge amount of troubles with that … for years’ (M3). In some cases, mothers' absence when travelling for CMC healthcare left siblings in parent‐like roles, caring for younger siblings when their father was working, saying ‘I just felt like I was sort of thrown into a role that a seven‐to‐eight‐year‐old shouldn't be in’ (S3). Another sibling described how he felt when his mother was away with CMC:

It was like empty … that just feels like empty, like we're [Dad and Sibling] alone. … If there's no one [Child] who messes something up … we won't be happy to clean up, because there'll be nothing to clean up. (S1).

Many siblings took pride in their caring roles, which instilled ‘a sense of purpose and‐‐ it made her feel good that she can help and make a difference’ (M10).

Extended family members often supported the family with sibling childcare or travelled to tertiary hospitals to provide direct support for mothers, often taking leave from work. CMCs' grandparents sometimes ‘uprooted her entire life to come and help’ (M1), or ‘retired so that … they've got a free life now where they don't have to take time off work. They can come … for weeks or months’ (M12) to provide ongoing support for the family. Other extended family members:

Used a lot of annual leave to go down when [Child]'s had his [therapy] intensives … I do feel really burnt out at the moment‐‐ it's probably just hit me a little bit with not taking leave … [for] what you should use it for (A12).

### 3.6 Family Relationships

Prior to receiving care coordination, many families described sibling attachment and connection issues as a result of frequent unexpected family separations: ‘we could leave in the middle of the night and then I was in [City] for two weeks … we definitely noticed a big change in her attachment … [she] would panic any time that I would leave’ (M9). Some parents expressed their perception that receiving care coordination early on in their experience of caring for CMC could have reduced the amount of unnecessary travel, making that period ‘a lot less disruptive on the whole family as a unit’ (M4). One CMC highlighted the need for support to enable parents to spend valuable time with siblings, stating that ‘what my family needs, family time … You [Mum] never have time with [Sister] and [Brother]’ (C3). As a father described, the care coordinator ‘being able to come in and help with alleviating that stress and burden has definitely helped us put our attention more towards‐‐ just like normal family things’ (F9).

### 3.7 Enabling Support Within and for Families

In their key caring roles, mothers relied significantly on support from fathers, with one mother saying: ‘I need him [Husband] at home from the moment he finishes work’ (M13). They also depended on support from siblings, extended family members, and ‘our beautiful friends’ (M8). Receiving care coordination enabled CMC to receive quality care closer to home (see Theme five), and this in turn enabled greater family presence and support for mothers. One mother highlighted this as valuable: ‘being closer to home [during Child hospitalization] that way my husband could help out and my mother‐in‐law could help out’ (M9).

Having multiple family members receive tertiary hospital training before CMC discharge was enabling and valuable for parents. In a family who received care coordination before their discharge home, both parents and also CMC's grandmother ‘went through all the training things as well’ and so she ‘could sit with her [Child] while she slept, so we [parents] could get some sleep, was worth its weight in gold’ (M5). This training and education involved learning essential skills to safely and effectively use and maintain medical equipment at home, such as feeding tubes or dialysis equipment. Without care coordination, training was typically only provided to mothers, which intensified their responsibility for providing care when returning home. The responsibility of teaching additional family members before they could help with care for CMC also fell to mothers, although one mother recognized the limitations of this approach, as she couldn't ‘teach you like those nurses who teach five people a day and actually know what they're doing’ (M1). This created a precarious situation described as being ‘on a knife edge because it's sort of relying on mum. Like if she gets crook [unwell] and there's something then well, we don't know what to do’ (F1).

As young carers, siblings supported parents by assisting in daily care tasks including bathing and playing with CMC, timing seizures, practicing therapy exercises, assisting with dialysis preparations, helping mothers remember health information for CMC, and in the case of older siblings, driving CMC to healthcare appointments. However, several families described insurmountable barriers to accessing young carer support for siblings, such as being unable to coordinate appropriate transport for siblings to attend a ‘fun day out’ (M13) through a carer support charity, or difficulty meeting eligibility requirements for young carer support.

Families gratefully acknowledged the support for temporary accommodation they received from the charity accommodation organisation Ronald McDonald House, which was facilitated by the care coordinators during tertiary hospital admissions for CMC, in some cases. Families particularly noted the value of having this support extended for multiple family members to stay together, with one CMC saying that ‘if Dad and [Brother] came up they could stay in Ronald MacDonald House as well. So that was really good’ (C6). Families also appreciated being ‘able to stay in the same room’ (M5) at their local hospital, as part of a coordinated discharge from the tertiary hospital, before returning home. However, several families discussed difficulties accessing accommodation support, for both fathers and extended family members, prior to receiving the care coordination service. Some fathers ‘had to find accommodation… myself while [Mum] and [Child] are in hospital’ (F9). In another case, one CMC's aunt and grandmother ‘actually ended up sleeping in [aunt's] car’ (A12) because they were unable to stay in the hospital or find other accommodation. This issue was also highlighted when charity accommodation staff could only offer ‘one room for a family kind of thing and I'm like, “well then, how does that work for parents that are co‐parenting?”’ (F11).

### 3.8 Quality Care Closer to Home

Care coordinators facilitated quality care closer to home by coordinating CMCs' discharge home from metropolitan hospitals, advocating for CMC during emergencies, and creating shared care plans. Healthcare emergencies were frequent for many CMC, and almost all families shared stories of challenges advocating for optimal healthcare for their children in rural settings, before receiving care coordination. Local emergency staff in rural hospitals did not typically have the experience or knowledge required to appreciate CMCs' specialized care needs. For example, one mother described ‘the amount of times I've jumped in to stop them … I'm like “yes, but her threshold is [CMC threshold] and you are treating at [standard threshold]”’ (M1). Critically, staff in local emergency departments (EDs) frequently failed to acknowledge parental expertise regarding CMCs' unique needs, with numerous families describing being ‘never heard … I just can't get people to listen’ (M12). The high staff turnover in rural health services meant limited expertise with the complex needs of CMC and concerns from parents about care quality being ‘just questionable, just hard’ for parents ‘dealing with’ (M9) healthcare professionals.

To overcome these challenges and their concerns for CMCs' safety, many families described developing ways to bypass EDs and instead contact their local paediatric staff directly, to advocate for them (Table 6). One parent highlighted: ‘you got to get to the right person and then things fall into place’ (F9). In contrast, one family described the benefits of local ED staff receiving detailed training in CMC's unique needs during a coordinated discharge process, and how care information for CMC was communicated, despite staffing changes, through ongoing use of a shared care plan (Table 5).

**Table 5:** Families' varied experiences with rural EDs.

Narrative	Quotes
Parental expertise regarding needs of CMC was denied repeatedly. After numerous negative experiences, parents learned that they needed to ensure they had advocacy support from the local paediatric team, to keep CMC safe.	F1: ‘We'd often come into ED and go … take us serious now please because we're not just worried parents we've got a bit of history … we know our kid better than anyone in this hospital … M1: We had started to call the pediatrician … and they would come and meet us because they knew we were getting brushed off … like a few times … we were like, ‘She's [having a medical emergency]’. And they're like, ‘Oh no she's not’ … And I was calling [Tertiary hospital] and clearly we couldn't get across there [due to COVID‐19 border restrictions]. So they're [tertiary hospital staff] going ‘go to [local ED]’ and I'd go there and they're like, ‘She's fine. Go home’. So we were in this cycle and then eventually we did get sent to [Tertiary hospital] and she had … more [tissue damage] … which was the final pin in that we learnt to call [local paediatric team] to tell them we were coming.
As part of the family's coordinated discharge from the tertiary hospital back to their home, the local ED received training regarding CMC's needs. During subsequent emergencies, the parents' caring expertise was fully acknowledged by hospital staff. They were treated like nurses and were free to manage their child's care during their hospital stay, and the staff and parents shared mutual confidence and respect.	M5: ‘Part of our discharge plan was … the staff at [Place] Hospital had to go through training for … [Child]'s uniqueness … they wanted to be very aware of everything before … being discharged … And I think they did some with emergency … because every time we went to emergency afterwards, they were very aware of her … [Emergencies are] definitely scary, but everyone was already so aware of her, and after the first couple of times, they were very familiar with her. So generally, when we go to the hospital in [Place], we do all her medical care down there, because we've got such a good routine … F5: Yeah, yeah … They pretty much put us as a‐‐ nurses down there‐‐ as doing everything … M5: We are free to do what we feel is best for [Child] down there … we've got all the confidence in the world in [the hospital staff], and it feels like they have all the confidence in the world in us'.

Abbreviations: CMC, children with medical complexity; ED, emergency department.

Families reported important benefits from having care coordinators connect CMCs' local healthcare providers with specialist care directives, either through direct and open communication or shared care plans (Table 6). One parent explained the necessity of having care coordinators facilitate this connection by being:

Really good at saying [to local paediatricians] ‘can you please ring … and speak with the tertiary specialist about it just to confirm what [Child]'s boxes are that need to be ticked not what‐‐ just the [Major Hospital] … says as a broad umbrella term'. (M9).

**Table 6:** Family stories reflecting the value of care plans.

Narrative	Quotes
This family had history of negative experiences with local emergency care (see Table 5). In this case, complex care directives were accessed during an emergency because the care plan was used successfully.	[Care coordinator] has now done a big care plan that when you open her file it pops up … and it's definitely helped. … Last time … when we used the care plan and it was ‘call [tertiary hospital]’ and … [Specialist] was away … but she had in her notes ‘the next step is to do this, this, this, this, this’. And so it was a case‐ ‘so according to [Specialist]'s notes, like we're introducing [medication], we're reintroducing at this dose, start with a low dose, immediately go to this and then schedule a review with her’. That was implemented and it worked. (M1)
Having a care plan could have ensured quicker transfer from the rural hospital to the tertiary hospital, and reduced wait time for operation notes to inform emergency surgery.	The logistics of how this trip happened would have been different if had care plan ‐ had 2‐hr road transfer first, waited for 12 hr [at rural hospital], then was flown [to tertiary hospital]. The 12‐hr wait was ‘really, really traumatic’ … but if had had care plan then might have been flown straight away if care plan clearly stated child needs. (M7 ‐ Mumford survey) When we arrived in [Tertiary hospital] that night at 3:00 AM … they couldn't get hold of … the on‐call surgeon at [other tertiary hospital] … and they didn't want to go and open him [Child] up until they got the operation notes [from previous surgery]. And I could just see the serious repercussions of them not having a record … And it was a horrible repercussion ‘cause he [Child] was … screaming in agony for hours while we waited for this information to come across. (M7)

Because direct communication was not always feasible or efficient across healthcare sectors and facilities, due to lack of shared electronic medical records, care coordinators facilitated this vital communication of CMC healthcare needs by creating care plans (Table 6).

## 4 Discussion

These findings highlight rural healthcare challenges experienced by families caring for CMC, and the importance of personalized approaches to address these challenges according to the circumstances of each family system. We identified key contextual factors that influenced families' experiences, including the severity and nature of CMCs' diagnoses and families' experience with caring, in addition to their rural location. Our findings add to the limited literature describing varied experiences amongst multiple family members caring for CMC [21], and begin to explore how support needs vary according to each family member's caring role.

Previous evidence describes the stress of uncertainty families experience while coming to terms with the illness of a child [48]. Stories from almost all families in this study confirmed the need for support through their initial crisis period. This included support in understanding their child's illness [49], learning how to navigate the health system [50], and establishing caring roles and responsibilities within their family [51]. To reduce long‐term negative impacts on physical and mental health [52], families should be connected with supportive interventions, including care coordination, at the earliest stage of their CMC caring journey.

Health systems are under‐equipped to serve the needs of children with complex, multi‐faceted needs who live distant from specialist services [3]. Previous authors have highlighted gaps in effective communication to support families' readiness for discharge from hospital to home [53]. Our findings additionally describe the importance of timely care coordination to ensure rural healthcare services' readiness for CMCs' discharge home to rural areas, to address the inherent risks of this transition and promote CMCs' safety. There is a need, therefore, to prioritize the delivery of care coordination services in rural areas to ensure that the health system responds to the individual needs of each child and their family, irrespective of where they live.

Care coordination played a crucial role in ensuring optimal child health outcomes in rural areas by facilitating communication of complex care directives among tertiary specialist teams, local paediatricians, ED staff, and most importantly with families. Addressing communication gaps between healthcare teams has been identified as a key role of care coordination [21]. Stories emerging from the current study confirmed the increased stakes for CMCs' safety in rural health services, and the importance of care coordination and development of shared care plans to enable sharing of critical CMC health information, and avoid reliance on rural healthcare staff with often limited experience managing complex and rare diseases in emergency situations [54]. Paediatric models of care increasingly focus on family‐centred care and promote healthcare providers' acknowledgement of parental expertise regarding their children's healthcare needs [55]. However, our findings indicate that such models of care may not translate to the standard practice in rural EDs, and parents continue to face challenges in being recognized for their caring expertise [50]. Care coordinators serve a critical role in advocating for safe and appropriate care for CMC and their families accessing rural healthcare services.

While these findings support the delivery of care coordination to alleviate the impact of caring on whole families, we draw attention to additional avenues for family‐based support, which are beyond the remit of current paediatric care coordination services. Some integrated paediatric care coordination models have expanded to address families' unmet psychosocial needs, such as access to family counselling services [24, 56]. Our findings add evidence of additional family needs, such as improving fathers' and extended family members' access to accommodation during tertiary hospital stays, and access to flexible, family‐centred employment. Further investigation is required, and planned by the authors, to confirm the support needs of multiple family members caring for CMC, alongside mothers, and explore how future interventions might provide this support.

## 5 Strengths and Limitations

Elevating fathers, siblings and extended family members' caring roles is a key strength of this research, as these carers are commonly hidden in research discourse [57]. The first author's closeness to participants afforded deeper empathy and insight into families' circumstances, adding both strength and limitation, and was tempered by noting inconsistencies and omissions and portraying experiences contextually [58]. Additionally, variability in interviewers may have influenced participants' responses, although this was mitigated by joint debriefing with members of the research team after interviews [37].

There were methodological limitations in this study. Although conducting both individual and joint interviews would have better captured diverse perspectives within families, this was not feasible due to the time capacity of our study population, and as such some perspectives may have been omitted [59]. Additionally, mothers were the dominant speakers in the joint interviews and sometimes acted as gatekeepers, determining whether to invite their additional family members to participate, which may have contributed to under‐representation of other family members' experiences. To mitigate this in future research, fathers, siblings, extended family members and CMC themselves should be engaged more directly, using additional recruitment methods. These participants should be interviewed individually to better understand their perspectives, as planned by the authors for future studies. These limitations represent some of the challenges of conducting research with carers and families managing chronic illnesses, who may have limited time and capacity for participation. Future studies must acknowledge and adapt to these challenges.

Additionally, the time elapsed since the events shared and the interviews may have influenced family members' recall. The results reported here may have limited generalizability outside of Australia due to nuances of the specific healthcare context, particularly in rural areas.

## 6 Conclusions

Understanding the experiences of whole families caring for CMC in rural areas is a crucial step to developing and implementing supportive interventions to improve all family members' health and wellbeing outcomes. The findings from this study are relevant for future implementation of care coordination models, further expanding models of care to address broader family needs beyond care coordination. Care coordinators play a vital role in supporting families to address health system barriers created by inadequate connection and information sharing between local rural services and specialist metropolitan centres, limited acknowledgement of family carers' expertise in CMC's unique care needs, and reduced service availability and access in rural areas. There is a need for future research to further explore the experiences of multiple family members caring for CMC, and particularly the perspectives of fathers in their key caring roles and in their roles supporting mothers.

## Author Contributions

**Anneliese de Groot:** conceptualization, methodology, data curation, investigation, formal analysis, visualization, writing – original draft, writing – review and editing. **Karen Hutchinson:** conceptualization, methodology, data curation, investigation, formal analysis, supervision, visualization, writing – review and editing. **Amy Hickman:** methodology, formal analysis, supervision, visualization, writing – review and editing. **Raghu Lingam:** conceptualization, funding acquisition, formal analysis, investigation, supervision, visualization, writing – review and editing. **Jeffery Fletcher:** conceptualization, formal analysis, investigation, supervision, writing – review and editing. **Hayley Smithers‐Sheedy:** conceptualization, formal analysis, investigation, data curation, writing – review and editing. **Yvonne Zurynski:** conceptualization, methodology, supervision, formal analysis, visualization, writing – review and editing.

## Ethics Statement

Ethical approval for this study was obtained from the Sydney Children's Hospitals Network Human Research Ethics Committee (2021/ETH01055). All participants gave informed consent.

## Conflicts of Interest

The authors declare no conflicts of interest.

## Supporting information


Supporting File

## Data Availability

Research data for this study are not shared as they are part of the ongoing evaluation of RuralKidsGPS.
