# Supplementary material for: Care Coordination Experiences of Whole Families Caring for Children With Medical Complexity in Rural Areas
Source: Health Expect. 2026 Aug 3;29(4):e70793. doi: 10.1111/hex.70793 (PMC13431284; doi:10.1111/hex.70793)
Supplement: Supplementary file 1 — Supporting File [file HEX-29-e70793-s001.docx]

**Appendix A:**

Exemplar quotes for Figure 2 - Accessing appropriate care when living in a rural area

| Narrative | Quotes |
| --- | --- |
| **Home**: families must organise extensively to prepare for travel | We are really lucky that we have somewhere that we can stay in [City] so if we choose to go up the night before … but that in itself is a big thing too, ‘cause you gotta prep to be-- however many feeds for [Child] umm and all this extra stuff to pack and to do the things that--- to have a sleepover somewhere with [Child]. (M2)    [Husband] and I are constantly having meetings on how we can make the thing that we need to do that week super easy. We have to plan ahead. I have to have like the freezer pack meals ready to go for breakfast, lunch, and dinner. (M8) |
| Mothers usually travel with CMC, sometimes also take siblings | I've had countless phone calls with [Care Coordinator] when I've been in [City] trying to coordinate appointments and trying to get them to understand that I'm traveling with three kids. I’m having to prioritise between how many days they have off, I'm trying to get down there as quick as I can, I'm trying to get back as quick as I can. I've got three kids in tow. (M4)  Every time we went down [to tertiary hospital] it was just me that could go ... so it's just usually me, myself, and I looking after her down there which is--- yeah, which is hard. ‘Cause there's no break. (M9) |
| Mothers' fatigue due to intensive travel and multiple appointments, tests and procedures | It's very difficult … The emotional side of it all as well because it-- you get tired. You're tired of driving, the kids don't want to do it. (M4)  I got home and [Dad]’s like, ‘What happened?’ And I was like ‘just don’t talk to me, please’ ... I just was so done that day I was just ‘I'm done, nobody speak to me’. (M2) |
| Fathers usually share time between their job and sibling childcare | I was back home with our son. (F6)  It's [travel to appointments] very disruptive ... I have to take time off [work] to ... help out looking after our other daughter. (F9) |
| Traveling with medical technology can be difficult and create family stress | [CHILD] is tube fed … it's pretty regimented feed routines … we can't be flexible with ‘oh just eat in the car’ like we have to pull over, plug her in, mix the formula, all that sort of thing- at that time … which is fine except for when you're sitting in a traffic jam and going, ‘Are we going to get to pull over in the next 40 minutes?’ ‘No.’ ‘All right.’ (M1)    That is actually a big deal … such a big stress in the day. It's like ‘we're off schedule for the feeds’ and everything is just-- kind of cascading negatively as a result. (M8) |
| Families often require support from an NDIS support worker to address CMC needs during travel. Uncoordinated travel consumes excessive amounts of families’ allocated NDIS plan. | We do intensives [therapy] in [City] ... we are scheduled to go back down there ... which (pause) I am quietly crapping my pants about with this whole [mother’s injury] situation. One of our support workers is flying down to ... stay with us for, I think six days, where she’ll um do the majority of it with me and [Child]. (M13)  We had a support worker come with us. NDIS support worker. So we paid her for a 12-hour shift. Actually I think it was 13 hours all up that day that we paid her to come along. Yeah. Which you know when it’s $62 an hour. That's a big chunk of your 25 hours a week of support work [in NDIS plan]. (M1) |
| Families rely on public toilet facilities when traveling, which are not always available. | Incontinence-- so you got to change nappies. There's not disabled toilets suitable anywhere along the way. If you don't change it, it goes throughout our car. (M1) |
| Some CMC experience distress during long trips due to their medical condition and anticipating medical appointments | Child has autism and gets very upset on way to appointments wondering what is going to happen, anticipating pain. (M3 - Mumford survey)  Traveling with Child in the car is anxiety-producing. He cries inconsolably and it is horrible for all who are present. (M13 - Mumford survey) |
| **Hospital:** administration systems not designed to accommodate the distance families travel, or coordinate appointments for children seeing multiple specialists | [Child] sees heaps of people … crazy appointment schedules, like we had some that were one week apart in [City] … and then we had some that were a day, one was one day, one was the next day, or two days apart or something, and I was like ‘that is not workable.  I can't do that.’ (M2) |
| Telehealth: parents often responsible for determining whether appropriate and requesting telehealth appointments | [Hospital] ... they don't really understand I guess the difficulties of being regional-- like attending medical appointments. So when they send requests out to us being like ... ‘[Child]'s got an upcoming appointment for this time’ we're trying our best to see whether that can be done via telehealth or whether ... they need to do it in person. ‘Cause they- they don't really send out saying ‘it's usually by telehealth’. They're just saying [Child] needs ... to be [reviewed]. (F9) |
| Some families need to stay overnight because the distance is too far to travel home immediately | As reported in results section of manuscript: Families lived distances of 100 - 499 kilometers away from their tertiary hospital (median: 200-299) |
| Other families may have the capacity to choose to stay overnight when traveling for outpatient appointments. | We always go up and stay at least one night because it's just too far to drive up and drive back [in one day] with [Child]. We tried it a few times and it was so stressful--- umm yeah so-- and even when we go up, we're going to stay two nights because he needs to have his sleep. (M8) |
| This requires them to organise accommodation that meets specific CMC needs. | Had to stay at [Hotel] which seems swanky but it's the only place that meets all their needs - accessibility, facilities. Very expensive and don't have a choice. (M12 - Mumford survey)    Trying to organise accommodation … close to the hospital given that when we go down … we don't drive down, we don't have … a car-- we're having to try to stay somewhere within walking distance to the hospital which was … a lot more difficult when [Child] was on oxygen ‘cause we had to, you know, transport the oxygen and umm-- all that equipment and you know, trying to walk from where we're where we were staying to the hospital. (F9) |
| For some families the cost of appropriate accommodation may be too high and they make long day trips. | You get the text message of ‘you will be in [City] at this time’ and you try and go, ‘8.15 doesn't work for me, I'm three hours away.’ And they go, ‘Oh well that clinic is full. Can you stay up the night before?’ … ‘Are you going to pay my $200 accommodation or am I leaving home at 4.30 in the morning?’ (M1) |
| Accommodation support doesn’t always meet needs of different family structures during hospitalisations, creating additional accommodation costs for second parents to be at the hospital. | There was like a week where I was able to stay in the Ronnie Mack House and then they were like, ‘no, it can only be one room for a family’ kind of thing and I'm like, ‘well then, how does that work for parents that are co-parenting?’ … and I didn't have money to be able to book a motel … 1500 bucks to book out a week kind of thing … unless you've got family like within 10 minutes of the hospital, you don't really have much of a choice but to either sleep in your car or (laughs) **…** try and get a room. (F11) |
| Many families experience financial difficulty due to the time they need to take off work to travel for CMC care. | I’ve returned to much less hours than I had intended to so therefore you accrue [leave] slower ... we’ve been doing this for two years now. At the moment it’s okay, but at the rate we’re going it’s not going to be okay in the long run. (M13)  I've gotta take a full day off work. His mum's gotta take a day off work so it's- you know- the- the money cost of driving up there, driving back, lunch, you know- food for Child--- all that kind of stuff- it- it all adds up and it all costs an absolute fortune at the end of it. (F11) |
| Families need financial support for travel costs but are intimidated by complicated applications processes. | I don’t wanna do the IPTAAS stuff because the forms and everything are all too painful-- everything is so overwhelming every time we do it [travel] that I just can't deal with that once we've gotten home and everything.’ (M2) |

CMC – Children with medical complexity, NDIS – National Disability Insurance Scheme
